# Highly Specific and Cost-Efficient Detection of *Salmonella* Paratyphi A Combining Aptamers with Single-Walled Carbon Nanotubes

**DOI:** 10.3390/s130506865

**Published:** 2013-05-22

**Authors:** Ming Yang, Zhihui Peng, Yi Ning, Yongzhe Chen, Qin Zhou, Le Deng

**Affiliations:** The Co-Construction Laboratory of Microbial Molecular Biology of Province Department and Ministry of Science and Technology, College of Life Sciences, Hunan Normal University, Changsha 410081, China; E-Mail: young02468@yahoo.cn (M.Y.)

**Keywords:** *Salmonella* Paratyphi A, aptamer, single-walled carbon nanotubes, detection

## Abstract

In this paper, a panel of single-stranded DNA aptamers with high affinity and specificity against *Salmonella* Paratyphi A was selected from an enriched oligonucleotide pool by a whole-cell-Systematic Evolution of Ligands by Exponential Enrichment (SELEX) procedure, during which four other *Salmonella* serovars were used as counter-selection targets. It was determined through a fluorescence assay that the selected aptamers had high binding ability and specificity to this pathogen. The dissociation constant of these aptamers were up to nanomolar range, and aptamer Apt22 with the lowest *K_d_* (47 ± 3 nM) was used in cell imaging experiments. To detect this bacteria with high specificity and cost-efficiently, a novel useful detection method was also constructed based on the noncovalent self-assembly of single-walled carbon nanotubes (SWNTs) and DNAzyme-labeled aptamer detection probes. The amounts of target bacteria could be quantified by exploiting chemoluminescence intensity changes at 420 nm and the detection limit of the method was 10^3^ cfu/mL. This study demonstrated the applicability of *Salmonella* specific aptamers and their potential for use in the detection of *Salmonella* in food, clinical and environmental samples.

## 1. Introduction

*Salmonellosis*, as we know, is one of the most frequently reported bacterial foodborn illnesses and is a significant worldwide economic and public health issue [1]. Among more than 2,500 serovars of *Salmonella enterica*, antibiotic-resistant *Salmonella enterica* serovar Paratyphi A (*S.* Paratyphi A), the agent of paratyphoid A fever, poses an emerging public health dilemma in endemic areas of Asia and among travelers [2], and it is presumed to cause less severe enteric fever than does *Salmonella enterica* serovar Typhi (*S. typhi*), so there is little research into *S.* Paratyphi A and no promptly available tolerable vaccination [3]. Rapid and precise identification of *S.* Paratyphi A as well as other specific *Salmonella* serovar from infected samples is critical for the subsequent treatment of infectious diseases caused by *Salmonella*. As the traditional bacterial detection method, plate incubation often has the limitations of lacking accuracy and being time-consuming. Modern popular rapid *Salmonella* detection methods such as enzyme-linked immunosorbent assay (ELISA) and PCR-based techniques have been developed. However, most methods cannot reach low detection limits without enrichment culturing and could lead to false positive reactions in *Salmonella* of different serovars. Due to their high affinity and specificity, aptamers have emerged as novel detection probes in the diagnosis of pathogens.

Aptamers are synthetic single-stranded oligonucleotides–either DNA or RNA–that can fold into a three-dimensional structure to bind specifically to a variety of targets, such as proteins, organic molecules, various cell surface receptors, and whole cells [4–8]. They are selected from a large pool of oligonucleotide (10^13^–10^15^ unique sequences) by an iterative approach called Systematic Evolution of Ligands by Exponential Enrichment (SELEX) [9,10]. Due to their high specific affinity, they are similar to chemical antibodies having low dissociation constants of nanomolar even picomolar level. Aptamers have several unique characteristics that make them more attractive in the fields of biosensing, diagnostics, therapeutics, and bioanalytical applications than antibodies. First, they can be selected in a time frame of 2 or 3 months by *in vitro* SELEX procedures without having to use cell lines or animals. Second, once selected, they can be easily and inexpensively synthesized or amplified *in vitro*. Third, unlike antibodies, they are low-molecular-weight, low-to-no toxicity or immunogenicity as well as higher physicochemical and biochemical stability. Finally, according to different purposes, they can be labeled with different functional groups [11].

SELEX was first reported in 1990 as a technique for screening aptamers. In the procedure, the method of isolating the bound DNA/RNA from the library is the most crucial step to ensure purity and selectivity. A series of separation techniques have been developed to increase the efficiency and throughput of aptamer isolation in last decades, such as capillary electrophoresis, flow cytometry [11]. Usually, a perfect aptamer should be selected in about 10–15 cycles and this takes months, so increasing the speed and simplicity of production are necessary. The Krylov group had developed a Non-Equilibrium Capillary Electrophoresis of Equilibrium Mixtures (NECEEM)-based non-SELEX approach, which can screen high-affinity aptamers with three rounds of selection and takes only 1 h [12]. Combining magnetic bead-assisted SELEX with microfluidics technology, the Soh group developed M-SELEX method, which used a designable microfluidic chip to fleetly produce aptamers [13].

Aptamers are also ideal tools for the detection of food-borne pathogens, especially bacteria, in environmental, food or clinical samples. Heretofore, few studies have reported aptamers against *Salmonella*, such as DNA aptamers against *S. typhi.* outer membrane proteins (OMPs) [6], RNA aptamers of *Salmonella enteritidis* [1]. However, aptamers against *S.* Paratyphi A have not yet been reported. In the present study, we have generated a panel of interesting ssDNA aptamers specific to *S.* Paratyphi A cell by using whole-cell-SELEX(WC-SELEX) [14]. These aptamers are selected by using whole intact cells as targets, while four other *Salmonella* serovars are used as negative control for the selection. Whole-cell-SELEX is favored in some particular cases, when the clear marker target is unknown. Aptamers generated from whole living cells are excellent molecular recognition probes to characterize target cells on a molecular level. These aptamers are effective for recognizing the target molecules such as cell-membrane receptors in their native conformations and physiological environments in a living cell, thus, it makes them an effective tool for biomarker discovery and molecular medicine [15].

Recently, great efforts have been made to combine the specific molecular recognition ability of aptamers with unique photophysical and structural characteristics of inorganic nanomaterials in the field of biosensors [16–21]. The carbon nanotubes, as one of excellent nanomaterials, have been most extensively studied as a result of their unique chemical, electrical, and mechanical properties [22–24]. Recently, some studies demonstrated that carbon nanotubes can protect ssDNA while they were delivered into intracellular systems via a self-assembly method, ssDNA are able to wrap onto single-walled carbon nanotubes (SWNTs) by π-stacking interactions between the nucleotide bases and the dispersion and separation of SWNTs will be enhance [25–28]. These unique properties make SWNTs become a new type of analytical tool. Also, the examples of noncovalent interactions of aptamers with SWNTs for the detection of biomolecules have already been reported [29–31].

**Figure 1.**
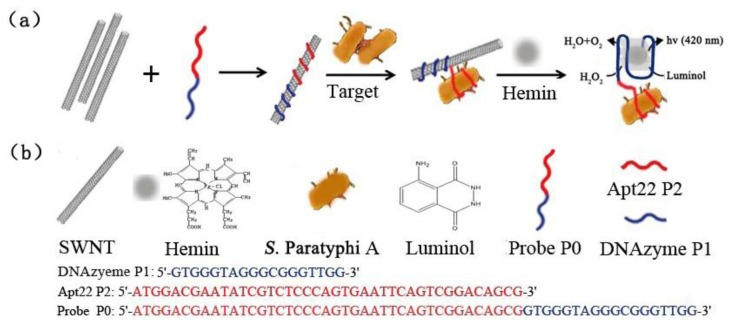
The detection method based on the noncovalent self-assembly of SWNTs and DNAzyme-labeled aptamer probe. (**a**) Scheme for detecting *S.* Paratyphi A. P0 wrap onto SWNTs to be a stable P0/SWNTs complex, then the target bacteria and hemin were added. P2 oligonucleotides specifically bind to target first and resulting in P0 keep away from SWNTs completely, and then a self-assembled formation of hemin/G-quadruplex horseradish peroxidase mimicking DNAzyme. And this self-assembled DNAzyme act as a catalyst for the generation of chemiluminescence (*λ* = 420 nm) through the oxidation of luminol by H_2_O_2_, which results in an amplification of the detection signal. This signal will be captured by fluorescence spectrometer. (**b**) Structures of Hemin, Luminol, SWNT, *S.* Paratyphi A, and oligonucleotides sequence of Probe P0, DNAzyme P1, and Apt22 P2.

Therefore, in this report, we present a specific and cost-effective sensing platform for detecting *S.* Paratyphi A in a culture medium, which is based on the noncovalent self-assembly of individual SWNTs and DNAzyme-labeled aptamer detection probe. In this approach, a DNAzyme sequence (P1) and aptamer Apt22 (P2) were chosen and designed as the detection probe P0 (Figure 1). The DNAzyme sequence was used as a label to amplify detection signals. Apt22 was selected because of its high binding affinity to the bacteria during the preliminary screening. Figure 1 shows the signaling scheme of this approach. Briefly, the probe P0 bound to individual SWNTs and resulted in a stable P0/SWNTs complex. Without the presence of target, the noncovalent hybridization of P0 with SWNTs can interfere the formation of any free hemin-containing active DNAzyme and the system would not generate detection signals. Upon adding the target bacteria and hemin, they will specifically bind to the probe and compete with the individual SWNTs [27], resulting in P0 keep away from SWNTs and a self-assembled formation of hemin/G-quadruplex horseradish peroxidase mimicking DNAzyme [32]. And this self-assembled DNAzyme act as a catalyst for the generation of chemiluminescence (*λ* = 420 nm) through the oxidation of luminol by H_2_O_2_ [33], which results in an amplification of the detection signal. It is possible to detect target by fluorescence spectrometer. It should be noted that the selectivity of the method is chiefly determined by the capability of aptamer Apt22 specifically against the target bacteria.

## 2. Experimental

### 2.1. Chemical Reagents

The combinatorial ssDNA aptamer library containing a 40-base central random sequence has primer sites on both sides: 5′-GAATTCAGTCGGACAGCG-N_40_-GATGGACGAATATCGTCTCC C-3′. The FITC-labeled forward primer (5′-C_12_-FITC-GAATTCAGTCGGACAGCG-3′) and biotin-labeled reverse primer (5′-Bio-GGGAGACGATATTCGTCCATC-3′) were used in PCR to get the double-labeled DNA and measure the concentration of the ssDNA in fluorescence analysis. The biotin-labeled DNA after denaturation was incubated with streptavidin-coated magnetic beads (M-280 Dynabeads, Invitrogen, Carlsbad, CA, USA) for procuring the FITC-labeled ssDNA pools. The library, primers, DNAzyme sequence and other DNA oligonucleotides were synthesized by Sangon Biotech (Shanghai, China). SWNTs with 2.73 wt%-COOH and ≤1.5 wt% ash content were obtained from Timesnano (Chengdu, China). Hemin was purchased from Porphyrin Products (Logan, UT, USA). Other reagents were purchased from Generay Biotech Co. Ltd. (Shanghai, China). All the other chemicals were of analytical reagent grade and were used as received without further purification. Solutions were prepared with double distilled water (ddH_2_O).

### 2.2. Apparatus

The amplification of the ssDNA was performed with a PCR instrument (Mastercycler pro, Eppendorf, Hamburg, Germany). The Origin Pro 7.5 software and the online software mfold (http://mfold.r na.albany.edu), respectively, were used for calculating the dissociation constant *K_d_* and the analysis of the structural folding (secondary structure) of aptamer sequences. Absorbance measurements were performed on an ultraviolet spectrophotometer (Techcomp, Inc, Shanghai, China), and the concentration of the ssDNA was calculated by this ultraviolet spectrophotometer. The fluorescence intensity and chemiluminescence light intensity were measured by a fluorescence spectrophotometer (LS55, Perkin–Elmer, Hayman, Germany). The FC 500 MPL flow cytometry instrument was bought from Beckman Coulter (Brea, CA, USA), the LSM710 confocal microscope was purchased from Carl Zeiss Corporation (Jena, Germany).The Millipore water was produced by an Ultra-pure water system (Millipore, Boston, MA, USA).

### 2.3. Bacterial Strains

The clinical isolation strain of *S.* Paratyphi A (isolated from patients infected with *Salmonella*), *E. coli*, ETEC K88 (CVCC 216), *Staphyloccocus aureus* (CMCC (B) 26113), and *E. coli* TOP10 were obtained from the Basic Medicine Department of Hunan University of Traditional Chinese Medicine (Changsha, China). The classical strain of *Salmonella* Enteritidis (ATCC 13076), *Salmonella* Typhimurium (CMCC 50115), *Salmonella* Cholerae-suis (ATCC10708), and *Salmonella* Arizonae (ATCC 13314) were obtained from the Changsha Food and Drug Administration (Changsha, China). All the bacteria were grown at 37 °C in Nutrient-Broth medium (peptone 10 g/L, beef extract powder 3 g/L, NaCl 5 g/L) with shaking at 100 rpm. To prepare bacteria for study, the bacteria were cultured and harvested at an early log growth phase (OD_600_ of about 2.1). The number of bacteria were determined via a plate count method. The bacteria were got by centrifugation at 1,000 × *g* for 15 min at 4 °C, washed three times with physiological saline, and finally suspended in physiological saline.

### 2.4. SELEX Procedure

The WC-SELEX procedure for selecting aptamers against the bacteria was carried out as follows [7]: the ssDNA pools (2,000 pmol for initial round) suspended in 400 μL selection buffer were heated at 95 °C for 5 min in a selection buffer containing 50 mM Tris-HCl (pH 7.4), 100 mM NaCl, 5 mM KCl, 1 mM MgCl_2_, and 0.1% NaN_3_ and then snap-cooled on ice for 10 min. Bovine serum albumin (BSA; Sigma, Saint Louis, MO, USA) and a fivefold molar excess of yeast tRNA (Invitrogen) were added to decrease the binding background. The bacteria (5 × 10^7^ cells for the first round and 5 × 10^6^ cells for subsequent rounds) suspended in 100 μL physiological saline were incubated with the ssDNA pools at 37 °C for 45 min with moderate shaking. Separation of bound and unbound ssDNA sequences was done by centrifugation at 8,000 × *g* for 10 min and washing three times with 500 μL selection buffer (with 0.2% BSA) to remove unbound and non-specifically bound aptamers. The bound ssDNAs were eluted by heating at 100 °C for 5 min in 50 μL sterile ddH_2_O, centrifuged at 22,000 × *g* for 15 min, then supernatant was collected. The eluted ssDNAs were then enriched by PCR amplification. FITC-labeled forward primer and biotin-labeled reverse primer were used for PCR amplification (5 min at 94 °C, then 60 s at 94 °C 1 min at 48 °C, and 1 min at 72 °C, followed by 10 min at 72 °C). The PCR products were incubated with streptavidin-coated magnetic beads, and then denatured in alkaline solution, then the FITC-labeled aptamer pool was separated in preparation for the next round of SELEX. In order to obtain aptamers with high affinity and specificity, four rounds of counter-SELEX were performed sequentially after the tenth round of SELEX, the aptamer pool (60 pmoles) was incubated with four other *Salmonella* serovars (*S.* Enteritidis, *S.* Typhimurium, *S.* Cholerae-suis, and *S.* Arizonae) (5 × 10^6^ cells), respectively. Following this step, three rounds of SELEX were carried out again. The aptamer binding ratio was acquired by analysis of the quantity of ssDNA after the each round selection. As a rule, the SELEX procedure takes several months.

### 2.5. Cloning and Sequencing of DNA Aptamers

Following 13 rounds of SELEX and four rounds of counter-SELEX, the 17th-selection PCR products were subcloned into a pUCm-T vector with the TA cloning kit (Sangon Biotech, Shanghai, China). To link the pUCm-T vector and PCR products, the T4 DNA ligase was added to the solution containing PCR products and a vector and was then held at 16 °C for 12 h. Then the plasmid from the 17th round of DNA sequences was used to transform *E. coli* TOP10 (TOPO TA Cloning kit; Invitrogen) to amplify the PCR products. Plasmids extracted from a single colony were sequenced by Sangon Biotech. In addition, the analysis of the structural folding (secondary structure) of aptamer sequences was performed by using the online software mfold [4].

### 2.6. Determination of Equilibrium Dissociation Constants

The affinities of the selected aptamers against the pathogen were confirmed by a number of binding assays. A range of concentrations of FITC-labelled aptamers (ranging from 20 to 200 nM) were prepared in selection buffer and incubated with the bacteria (10^8^ cfu/mL) at 37 °C for 45 min with gentle rotation in 500 μL selection buffer. Binding assays for each concentration were performed in three independent trials and analyzed using the fluorescence spectrophotometer, and then the quantity of aptamers bound to their target were calculated. On the basis of these data a saturation curve was obtained and the dissociation constant *K_d_* was calculated by the equation *Y* = *B_max_X*/(*K_d_* + *X*) [4], *B_max_* is the maximal fluorescent intensity measured by this experiment, and *X* is the concentration of the aptamer that was added, and *Y* represents the mean fluorescent intensity.

### 2.7. Flow Cytometric Analysis and Cell Imaging

To determine if high-affinity aptamers are being enriched in a selected ssDNA pool or the binding capacity of the selected candidate aptamers, it is necessary to monitor the binding of pool or candidate aptamers by using flow cytometry. The FITC-labeled ssDNA pool (100 nM) or candidate aptamers (300 nM) were incubated with the bacteria (10^8^ cfu/mL) at 37 °C for 45 min with moderate shaking in 200 μL selection buffer. The fluorescence was determined with a flow cytometry by counting 10,000 events.

Imaging of the cells was performed by using a confocal microscopy. The selected FITC-labeled aptamer with high affinity (300 nM) was incubated with the bacteria (10^8^ cfu/mL) at 37 °C for 45 min with gentle rotation in 500 μL selection buffer. After washing three times, the cells were immobilized on glass slide by 3% paraformaldehyde, then observed under the confocal microscope.

### 2.8. Specificity Identification of Selected Aptamers

To demonstrate their specificity, the selected FITC-labeled aptamers (200 nM) were incubated with *S.* Paratyphi A, *E. coli* K88, *S. aureus*, *S.* Enteritidis, *S.* Cholerae-suis, and *S.* Arizonae (10^8^ cfu/mL) in 500 μL selection buffer, respectively. The binding affinities of the aptamers against these bacteria were obtained through monitoring the fluorescence intensity.

### 2.9. Based on the Self-Assembled Aptamer Probes with SWNTs Conjugates for the Detection of S. Paratyphi A

To sensitivily and cost-effectivily detect it, DNAzyme-labeled aptamer detection probe P0 and SWNTs were combined. The Hipco process SWNTs with –COOH content were first sonicated in DMF for 30 min to give a homogeneous black solution, and then diluted with working solution I (25 mM HEPES, 20 mM KNO_3_, 200 mM NaNO_3_, 0.025% Triton X-100, 1% DMSO, pH 8.0) to make SWNTs suspension. The fresh SWNTs suspension was incubated with P0 (500 nM) at 37 °C for 4 h. After this step, the solution was centrifuged at 22,000 × *g* for 15 min and the supernatant containing the P0/SWNTs complexes was collected [22]. In order to avoid false positive, it is necessary to survey the ratio of P0 to SWNTs. The absorbance of the working solution containing P0 (500 nM) and different concentrations of SWNTs were studied during the incubation procedure. And then the target and hemin (1.25 μM) were added to the supernatant to react at 37 °C for 15 h. Following luminol (0.5 mM) and H_2_O_2_ (30 mM) were quickly added to the mixture solution, and the light emission intensity was measured immediately with the fluorescence spectrophotometer. In addition, the specificity of P0/SWNTs toward target was also tested, four other bacteria were tested as control experiments at the same conditions.

### 2.10. City Water Sample Analysis Based on the Detection System

City water was used as matrix for the detection of *S.* Paratyphi A in the environment. The water samples (500 μL) spiked with bacteria with different concentrations were prepared in centrifuge tubes, and then P0/SWNTs complexes solution (500 μL) prepared in working solution II (50 mM HEPES, 40 mM KNO_3_, 400 mM NaNO_3_, 0.05% Triton X-100, 2% DMSO, pH 9.0) and hemin (1.25 μM) were added and reacted at 37 °C for 15 h. As soon as luminol (0.5 mM) and H_2_O_2_ (30 mM) were added to the mixture solution, the light emission intensity was measured immediately. As for specificity, four different bacteria were added to the system and detected under the same condition.

## 3. Results and Discussion

### 3.1. WC-SELEX for Evolution of S. Paratyphi A-Specific Aptamers

In this *in vitro* WC-SELEX procedure, DNA aptamers, binding to the bacteria with high affinity and specificity, were screened from a random sequence library of 4^40^ DNA molecules. The initial aptamer library was comprised of 79 nt ssDNA, which included random 40-mer nucleotide inserts. The aptamers had a strong affinity for this pathogen after 13 rounds of selection and four rounds of counter selection.

**Figure 2.**
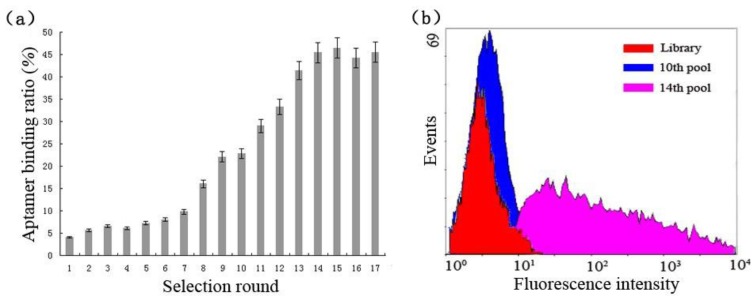
Enrichment of S. Paratyphi A-specific aptamers during the whole-cell SELEX procedure. (**a**) The bar graph reveals the ratio of the amounts of ssDNA eluted to the amounts of ssDNA pool in this round selection. Error bars indicate standard deviation (n = 3). (**b**) Flow cytometry assay to monitor the pool enrichment for *S.* Paratyphi A.

The enrichment of cell-specific aptamers was calculated during the selection process, the ratio of aptamers against target cells was less than 10% in the first six rounds (Figure 2(a)), then had a steady increase from round 7 to round 9 and reached to 22.1%, and no more at round 10. In four rounds of counter selections, the ratio value increased rapidly and reached to as high as 45.4%, and then kept a steady state from round 15 to round 17. It implied that the specific aptamers were saturated and successfully enriched as well. To further monitor the enrichment of the cell-specific aptamers during the whole-cell SELEX procedure, we employed flow cytometry to test the aptamers binding for suspension cells. As shown in Figure 2(b) with increased numbers of selection cycles, the ssDNA aptamers with better binding affinity to the target cells were enriched in the first ten rounds. In flow cytometry analysis, an increasing fluorescence intensity on bacteria was observed, and a significant change was shown in the fourteenth round. These results illustrated that the ssDNA pool was successfully enriched for cell-specific aptamers which bind to target cells and the counter-SELEX maintained the aptamer specificity in the process. After 17 rounds of selections, the purified PCR products of the last eluted ssDNA aptamers were cloned into *E. coli* TOP10 cells using the pUCm-T vector System, then the vectors containing intended fragments were sequenced in both directions with an ABI-PRISM3730 automated sequencer (Applied Biosystems, Foster City, CA, USA) by Sangon Biotech.

### 3.2. Identification of Selected Aptamers for S. Paratyphi A and Tests of Binding Ability

100 clones were sequenced in this experiment. The secondary structure and homology of these sequences were analyzed using the internet-based tool mfold and the software-DNASIS MAX, respectively. Analysis of the selected aptamers revealed that they could all be divided into four families based on their sequence homology and many repeats were observed in each family. The aptamers Apt10, Apt22, Apt45, Apt60, which were randomly chosen as the agent of each family, were used for further characterization (Table 1).

As shown in Table 1, the binding affinities of the aptamers were determined by using the fluorescence spectrophotometer. Their *K_d_* values were calculated and up to nanomolar range, in which the family II have the lowest *K_d_* (47 ± 3 nM). After being sequenced, it was found that the sequences of some aptamers were longer or shorter than the initial library; such as, the length of the family I aptamers were 3 or 4-mer more than the original sequence and the family IV aptamers sequences were 2 or 3-mer less than the original sequence.

### 3.3. Flow Cytometric Analysis of the Binding Capacity of Selected Aptamers and Cell Imaging

**Figure 3.**
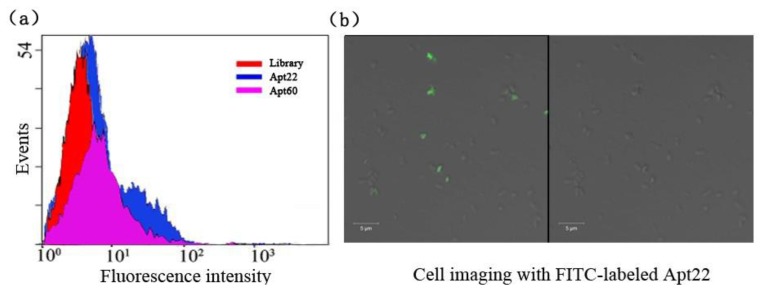
(**a**) Flow cytometric analysis of the binding capacity of selected candidate aptamers Apt22 and Apt60, the library as the control. (**b**) Confocal imaging of cells stained by the aptamer Apt22 labeled with FITC, left is the fluorescence image and right is optical image.

The selected aptamers Apt22 and Apt60 with low *K_d_* were picked to illuminate the binding capacity to *S.* Paratyphi A via flow cytometric analysis, the library as the control (Figure 3(a)).

After being incubated with the target bacteria, the binding of FITC-labeled aptamer Apt22 with the bacteria were monitored by flow cytometry. It showed that 27.9 % bacteria were detected by counting 10,000 events. But it was 26.5% of aptamer Apt60 in the same condition (data not shown). It indicated that the aptamer Apt22 had a higher binding capacity to *S.* Paratyphi A than aptamer Apt60. In this experiment, the highest binding affinity aptamer Apt22 was labeled with FITC and incubated with target for cell imaging under the confocal microscope (Figure 3(b)). Many cells presented bright fluorescence (green) on the periphery.

### 3.4. Specificity Identification of Selected Aptamers

**Figure 4.**
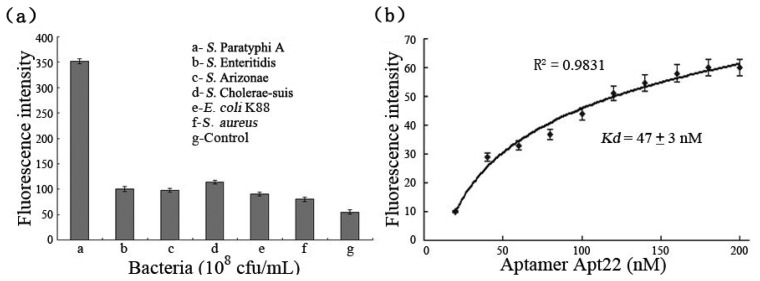
(**a**) The fluorescence intensities of *S.* Paratyphi A, *S.* Enteritidis, *S.* Cholerae-suis, *S.* Arizonae, *E. coli* K88, and *S. aureus* (10^8^ cfu/mL) bound to FITC-labeled aptamer Apt22 (200 nM). (**b**) Using the fluorescence spectrophotometer to determine the binding affinity of the FITC-labeled aptamer Apt22 to *S.* Paratyphi A. Error bars indicate standard deviation (*n* = 3).

To provide evidence of the binding specificities of the selected aptamers to target bacteria, the excessive FITC-labeled aptamer Apt22 (200 nM) was incubated with *S.* Paratyphi A, *E. coli* K88, *S. aureus, S.* Enteritidis, *S.* Cholerae-suis, and *S.* Arizonae (10^8^ cfu/mL), respectively. Control experiments were carried out at the absence of the bacteria. In fact, the detection signal (the fluorescence intensity subtracted the control signal) of *S.* Paratyphi A bound to FITC-labeled aptamer Apt22 was five-fold than the detection signals of other bacteria at least, and the nonspecific adsorption between aptamer and bacteria was not included (Figure 4(a)). The results displayed that the selected aptamers had a high selectivity and specificity against *S.* Paratyphi A, and the aptamer Apt22 was specifically bound to target bacteria without any cross-reactivity to other *Salmonella* serovars and pathogens. It indicated that the aptamer Apt22 could be used as an alternative screening tool in the detection of *S.* Paratyphi A. The binding affinity of the FITC-labeled aptamer Apt22 to the bacteria was calculated by using the fluorescence spectrophotometer (Figure 4(b)). The dissociation constant *K_d_* (47 ± 3 nM) of aptamer Apt22 was calculated by using the Origin Pro 7.5 software to draw the saturation curve (R^2^ of 0.9831).

### 3.5. Based on the Self-Assembled Aptamer Probes with SWNTs Conjugates for the Bacteria Detection

To ensure the supernatant containing only P0/SWNTs complexes after incubating and centrifuging, the absorbance of the working solution containing P0 (500 nM) and different concentrations of SWNTs were studied during the incubation procedure (Table 2). When the concentration of SWNTs was up to 0.10 mg/mL, the absorbance of the working solution was not decreased any more, it demonstrated that the SWNTs were in excess and there was no free probe P0 in the supernatant. In the next experiment, the concentration of SWNTs was 0.10 mg/mL in working solution.

**Figure 5.**
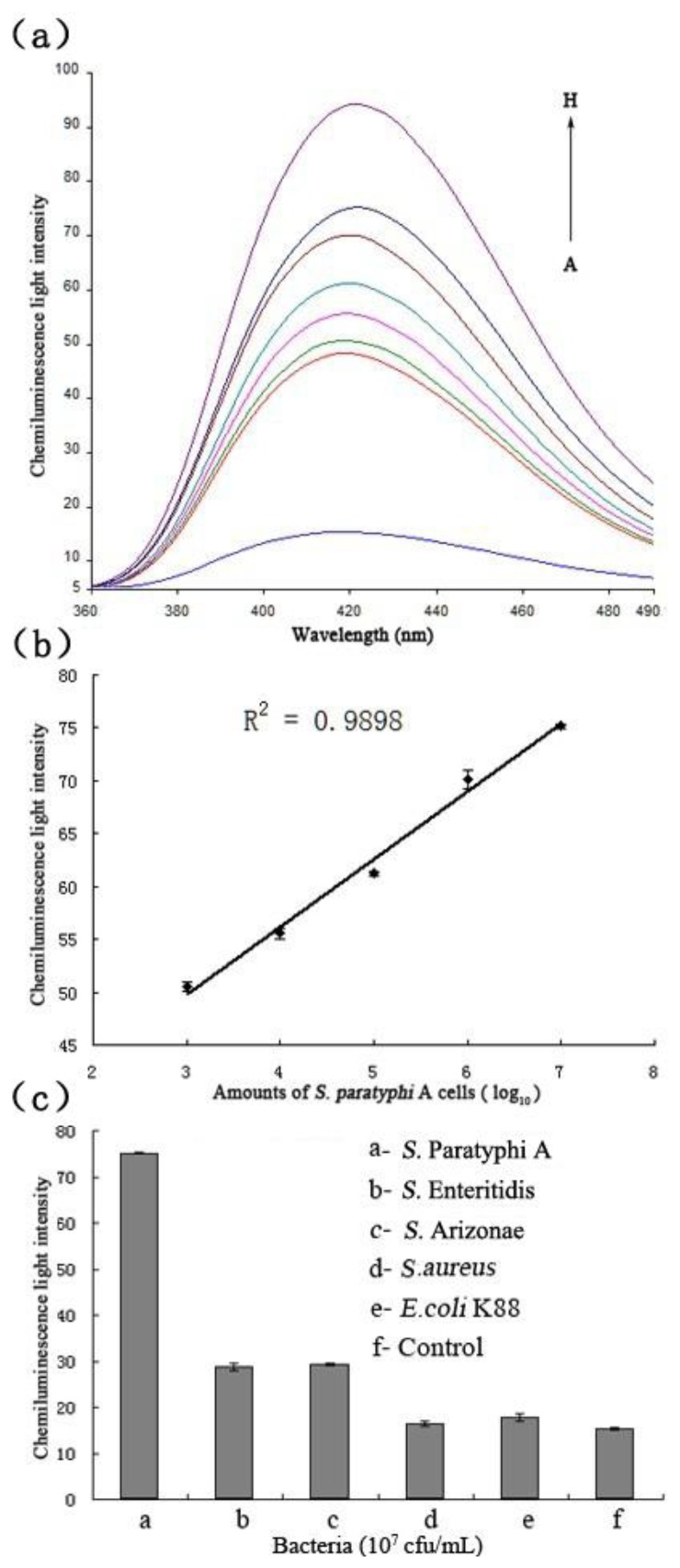
(**a**) Chemiluminescences of different amounts of *S.* Paratyphi A cells (A, 0; B, 10^2^; C, 10^3^; D, 10^4^; E, 10^5^; F, 10^6^; G, 10^7^; and H, 10^8^ cfu/mL). (**b**) The calibration curve of the chemiluminescence signal of *S.* Paratyphi A cells ranging from 10^3^ to 10^7^cfu/mL at *λ* = 420 nm. (**c**) The chemiluminescence intensities of target, *S.* Enteritidis, *S.* Arizonae, *S. aureus*, and *E. coli* K88 (a–e) were tested at the same conditions with the amounts of 10^7^ cfu/mL. f was the control. In all samples, [P0] = 500 nM, [hemin] = 1.25 μM, [luminol] = 0.5 mM, [H_2_O_2_] = 30 mM, in working solution I that included 25 mM HEPES, 20 mM KNO_3_, 200 mM NaNO_3_, 0.025% Triton X-100, 1% DMSO, pH 8.0. Error bars indicate standard deviation (*n* = 3).

The chemiluminescent principle of the detection system towards the target bacteria is displayed in Figure 1. As soon as the target and hemin were added to the solution, the hemin/G-quadruplex was quickly produced. The assembled DNAzyme act as a catalyst for the generation of chemiluminescence (*λ* = 420 nm) through the oxidation of luminol by H_2_O_2_, Figure 5(a) displays the chemiluminescence light intensity upon analyzing various amounts of target cells ranging from 10^2^ to10^8^ cfu/mL, and the resulting calibration curve in Figure 5(b). In the absence of target cells, the background signal of the control was displayed first (Curve A). With increasing amounts of *S.* Paratyphi A cells, the chemiluminescence light intensity increased (Curves B-H), which implied more dissociation of the P0/SWNTs complexes and a higher content of the DNAzyme. When the amount of *S.* Paratyphi A was 10^2^ cfu/mL, the generated signal was almost equal to 10^3^ cfu/mL. However, a linear range of chemiluminescence light intensities was observed ranging from 10^3^ to 10^7^ cfu/mL (R^2^ of 0.9898). It was showed that the detection limit of target bacteria was 10^3^ cfu/mL. The specificity of the detection system was also tested (Figure 5(c)). All the bacteria were tested under the same conditions in the amount of 10^7^ cfu/mL, respectively. Control experiments were done at the same time. The detection signals of target bacteria were 4.4-fold than *S.* Enteritidis, 4.3-fold than *S.* Arizonae, 56.3-fold than *S. aureus*, and 24.3-fold than *E. coli* K88.

The results clearly demonstrated that the detection system could detect *S.* Paratyphi A with high specificity and had no cross-reaction with other *Salmonella* serovars and pathogens. Importantly, it required neither the extraction of specific genes nor the precise probe design and synthesis [34]. Although the detection limits of some earlier methods were lower, their probes could be labeled with costly biotin [35], iuminophor [4], and conjugated with magnetic nanoparticles [36], or combined by using complicated quantitative real-time RT-PCR [6,37]. It must be also stressed that labeled aptamer probes and special instruments are not required in this method, which is more convenient and efficient to quickly complete the pathogen detection, and low-cost at the same time. Moreover, it could be potentially applied in principle to detect other bacteria by substituting the aptamer of *S.* Paratyphi A.

### 3.6. The Detection of S. Paratyphi A in City Water

**Figure 6.**
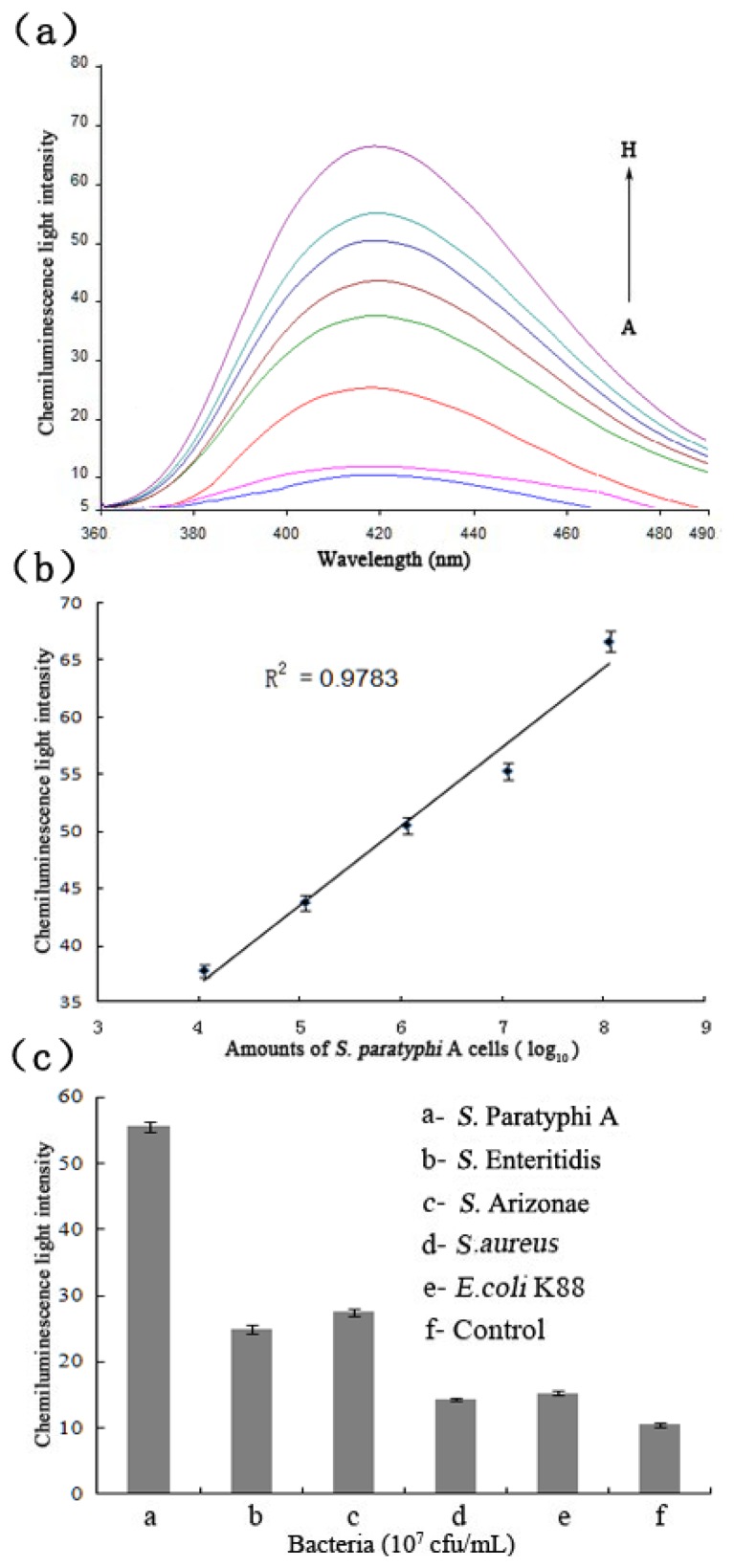
(**a**) Chemiluminescences of the city water spiked with *S.* Paratyphi A with different concentrations (A, 0; B, 10^2^; C, 10^3^; D, 10^4^; E, 10^5^; F, 10^6^; G, 10^7^; and H, 10^8^ cfu/mL). (**b**) The calibration curves of the chemiluminescence signals of *S.* Paratyphi A ranged from 10^4^ to 10^8^ cfu/mL at *λ* = 420 nm. (**c**) The chemiluminescence intensities of *S.* Paratyphi A, *S.* Enteritidis, *S.* Arizonae, *S. aureus*, and *E. coli* K88 (a–e) in city water were tested at the same conditions with the amounts of 10^7^ cfu/mL. Error bars indicate standard deviation (*n* = 3).

To demonstrate applicability of the selected aptamer, the city water spiked with bacteria with different concentrations (the final concentrations in the mixture solution ranging from 10^2^ to 10^8^ cfu/mL) were analysed by the detection system (Figure 6). When the amount of *S.* Paratyphi A was 10^4^ cfu/mL, the generated signal was more than thrice the control (Figure 6(a)), and a linear range from 10^4^ to 10^8^ cfu/mL (R^2^ of 0.9783) (Figure 6(b)). It indicated the detection limit of target bacteria in city water was 10^4^ cfu/mL. The specificity experiments were also tested (Figure 6(c)).

Four other bacteria spiked samples were tested at the same conditions with the amounts of 10^7^ cfu/mL, respectively. The detection signal of *S.* Paratyphi A was at least 2.7-fold more than for the other bacteria. Therefore, these experiments demonstrated that the selected aptamer could be also used to detect the real samples.

## 4. Conclusions

Aptamers have become one of the most popular research objects in the last decade, and have been used in a broader range of applications from biosensing to diagnostics to therapeutics and basic research. However, there are little reports about using aptamers directly against bacteria. To our knowledge, this is the first report of an aptamer selected for the detection of *S.* Paratyphi A and its specificities to other *Salmonella* serovars were also evaluated. This work presents a high affinity (the *K_d_* is down to 47 ± 3 nM) and specificity towards the target bacteria by screening from the WC-SELEX technology. Combining the use of carbon nanomaterials, a novel useful detection method based on the noncovalent self-assembly of SWNTs and DNAzyme-labeled aptamer detection probe was created. It can selectively and efficiently detect the pathogen with a detection limit of 10^3^ cfu/mL and a linear range of 10^3^ to 10^7^ cfu/mL, and a detection limit of 10^4^ cfu/mL in spiked samples. In conclusion, this work demonstrated the applicability of *Salmonella*-specific aptamers and their potential use in the detection of *Salmonella* in food, clinical and environmental samples by the development of aptasensors or kits.

## Figures and Tables

**Table 1.** Four families of aptamers selected via the SELEX procedure and the sequences and *K_d_* of their agents.

**Family**	**Aptamer Clone**	**Aptamer Agents**	**Sequence (from 5**′ **to 3**′**)**	***K****_d_*[**nM**]
I	11	Apt10	GATGATGGACGTATATCGTCTCCCATGAATTCAGTCGGACAGCG	73 ± 9
II	37	Apt 22	ATGGACGAATATCGTCTCCCAGTGAATTCAGTCGGACAGCG	47 ± 3
III	19	Apt 45	ATGGACGAATATCGTCTCCCAGTGAATTCAGTCGGACAGC	68 ± 6
IV	33	Apt 60	CGCCCACCCATAATGGATCAGGGCGGGCACCACGATG	56 ± 9

**Table 2.** The absorbance of the working solution containing probe P0 (500 nM) and different concentrations of SWNTs during the incubation procedure.

**The Concentrations of SWNTs in Solution [mg/mL]**	**Absorbance of OD_260_**
0.02	0.619
0.04	0.333
0.06	0.212
0.08	0.180
0.10	0.189
0.12	0.189
